# Covalently Linked Poplar and Ayous Lignin-Based Hydrogels: Sustainable Materials for Water Remediation

**DOI:** 10.1021/acsomega.5c10163

**Published:** 2026-01-27

**Authors:** Lamyea Yeasmin, Angelica Giovagnoli, Valentina Di Matteo, Stefano Scurti, Maria Cristina Cassani, Silvia Panzavolta, Asma Munir, Ilaria Ragazzini, Barbara Ballarin

**Affiliations:** † Department of Industrial Chemistry “Toso Montanari”, Bologna University, UdR INSTM of Bologna, Via Piero Gobetti 85, I-40129 Bologna, Italy; ‡ Center for Industrial Research-Advanced Applications in Mechanical Engineering and Materials Technology CIRI MAM, 9296 University of Bologna, Viale del Risorgimento 2, I-40136 Bologna, Italy; § Center for Industrial Research-Fonti Rinnovabili, Ambiente, Mare e Energia CIRI FRAME University of Bologna, Viale del Risorgimento 2, I-40136 Bologna, Italy; ∥ Department of Chemistry “Giacomo Ciamician”, 9296 University of Bologna, Via Piero Gobetti 83, I-40129 Bologna, Italy; ⊥ 19032 Politecnico di Torino, Corso Duca degli Abruzzi, 24 − 10129 Torino, Italy; # ALPI, Research Manager Viale della Repubblica 34, 47015 Modigliana, FC, Italy

## Abstract

Lignin-based materials are attracting increasing attention for their effectiveness in treating polluted water. In this study, special emphasis is placed on hydrogels modified with lignin extracted from Poplar and Ayous (Alpi Spa wood waste) which were developed for the removal of organic dyes, using methylene blue as a model pollutant. The two types of wood differ notably in their characteristics. Poplar is a medium-density hardwood with a relatively low lignin content, higher water repellence, and good ability to be shaped and glued. Ayous is a tropical wood; its lignin has a different chemical makeup, offering greater resistance to microbial degradation. However, Ayous is more prone to moisture absorption and less durable outdoors if untreated. The lignins covalently incorporated into polyvinyl alcohol (PVA)-based hydrogel via direct esterification with polyamide epichlorohydrin (PAE) were investigated. This chemical crosslinking strategy significantly reduced lignin leaching. The resulting hydrogels were thoroughly characterized by ATR-FTIR spectroscopy, thermogravimetric analysis, and scanning electron microscopy to elucidate their structure and morphology. Batch adsorption experiments revealed that hydrogels incorporating lignin from Ayous hardwood waste achieved remarkable methylene blue removal efficiency (up to 88%), a substantial improvement over both hydrogels incorporating commercial lignin (64%) and unmodified hydrogel (9%). By integrating lignin’s adsorption capabilities with the porosity and recoverability of the hydrogel matrix, the Ayous lignin-modified hydrogels present a highly promising and sustainable solution for eliminating organic pollutants from wastewater. This innovative approach not only enhances pollutant removal but also promotes environmental sustainability and aligns with circular economy principles.

## Graphical abstract

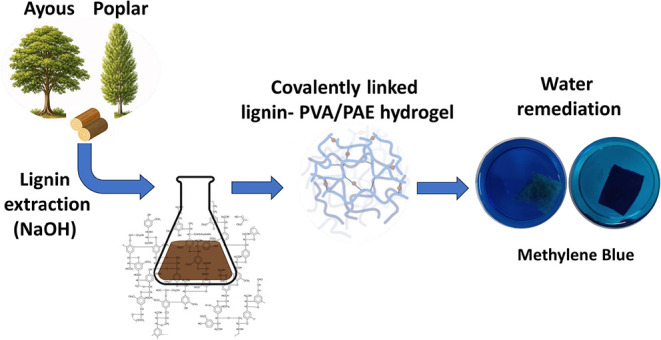

## 1 Introduction

Biomass-derived materials have emerged as a focal point in the development of advanced functional materials, with lignin standing out due to its cost-effectiveness and biodegradability.^1–3^ As an abundant, renewable resource, lignin is naturally broken down by microorganisms and their enzymes into valuable building blocks, which can then be leveraged for a variety of high-value applications.^4–6^ With the global pulp, paper, and wood industries generating over 50 million tons of lignin annually, this biopolymer is readily available and economically attractive thanks to its unique aromatic structure and low production cost.^7–11^ Lignin is a complex three-dimensional biopolymer containing randomly cross-linked phenylpropanoid units (i.e., coniferyl, sinapyl, and coumaryl alcohol). These monolignol units provide the base for the formation of lignin building blocks, i.e., p-hydroxyphenyl (H), guaiacyl (G), and syringyl (S).^12^ The proportion and type of these monomers, as well as the lignin building block, vary between species, resulting in differences in the chemical structure and mechanical and physical properties of wood.^11,13,14^ The composition of lignin varies across different sources and is influenced by the type of biomass, harvesting, and storage practices, as well as the geographic origin.

Recent years have seen considerable efforts to exploit lignin’s versatile chemical structure for the development of innovative materials for diverse applications,^14^ including as a reinforcing agent in composites,^15^ an antioxidant,^16^ a UV-blocking component,^17^ an antimicrobial additive,^18^ a battery binder,^19^ and in biomedicine.^20^ Increasingly, lignin recovery and valorization have become research priorities for environmental remediation, particularly in water treatment.^21–24^ This is intrinsically linked to lignin’s high chemical and mechanical stability, large specific surface area, environmental compatibility, versatility, and notable adsorption capabilities.^25^

Among the many valorization strategies, the integration of lignin into functional materials such as functionalized lignin particles, lignin–metal nanocomposites, and, notably, lignin-based hydrogels has proven especially promising for wastewater treatment applications.^26^ Lignin-based hydrogels are attracting attention as next-generation, sustainable adsorbents for organic pollutants.^26^ These hydrogels can be synthesized either by physically incorporating lignin into hydrogel matrices or, more effectively, by covalent chemical cross-linking to minimize lignin leaching, thereby enhancing stability and performance.^15,27–30^ The strategies commonly used to prepare lignin-based hydrogels consist in interpenetrating polymer network (IPN), cross-linking copolymerization, copolymerization of grafted lignin with hydrophilic monomers in the presence of cross-linker or atom transfer radical polymerization (ATRP), and reversible addition–fragmentation chain transfer polymerization (RAFT)^25,31,32^

Lignin-based hydrogels uniquely combine the plentiful adsorption sites of lignin with the intrinsically porous and highly recoverable structures of hydrogels. This synergy imparts these materials with high specific surface area, superior porosity, robust mechanical properties, and outstanding chemical stability, enabling efficient adsorption of pollutants via multiple interaction mechanisms, such as chemical bonding, electrostatic attraction, and hydrogen bonding. These features make them particularly suitable for removing organic dyes,^33–36^ which constitute a significant portion of industrial wastewater and are well known for their persistence and toxicity in aquatic environments and human health.^37–41^

In our previous work, we developed flexible, nontoxic hydrogels via a simple, cost-effective freeze–thaw approach for physical cross-linking.^42,43^ Building on these findings, this study focuses on lignin-modified hydrogels as advanced adsorbent materials for water remediation. The lignin was extracted from untreated industrial wood residue (Alpi Spa), specifically Poplar (Populus L.) and Ayous (Triplochiton scleroxylon), and compared with commercial alkali lignin. We chose these two woods for their different properties.^11,44^ Poplar is a medium-density hardwood with a relatively low lignin content, accounting for approximately 20–25% of the wood’s mass. Poplar lignin generally has a higher proportion of sinapyl units and the presence of a high proportion of S units, resulting in a more linear and less branched structure. This makes the wood lighter, easier to work with, and less resistant to moisture. Moreover, syringyl units have two methoxy groups (−OCH_3_) on the aromatic ring, while guaiacyl units have only one. This reduces the density of free phenolic groups in S-lignin, making it more hydrophobic and less chemically reactive, with a lower density of active sites for possible interaction with aromatic cations. This composition also contributes to its greater water repellence and good ability to be shaped and glued, which makes it ideal for the manufacture of furniture, plywood, and paper. Ayous is a tropical wood, botanically classified as a hardwood (broadleaf), because it comes from a broadleaf tree, not a conifer. However, from a physical and application standpoint, it is often considered softwood due to its lightweight and low hardness. For this reason, Ayous tends to have a lignin with a different chemical composition, often with a higher content of coniferyl units and more branched units in the lignin polymer consisting mainly of G units. This characteristic makes Ayous lignin more resistant to microbial attack, but at the same time, the wood is more susceptible to moisture absorption and less durable outdoors if untreated. It is widely used in applications requiring a fine finish and a good surface appearance.

This different lignin was covalently incorporated into a poly­(vinyl alcohol) (PVA) hydrogel network to prepare hybrid materials. The polyaminoamide-epichlorohydrin (PAE), a polyelectrolyte typically used as a wet-strength agent, commonly utilized in filter papers and paper towels,^45^ was used as a sustainable covalent linker.^46^ The lignin/PVA hydrogels were thoroughly characterized and assessed for their efficacy in the removal of methylene blue, a model cationic dye commonly present in industrial effluents and notorious for its toxicity.^47^ Lignin/PVA hydrogels were subjected to adsorption kinetic studies, revealing that the efficiency of dye removal is strongly influenced by the lignin source and the hydrogel structure itself. The workflow is summarized in Scheme 1.

**Scheme 1:**
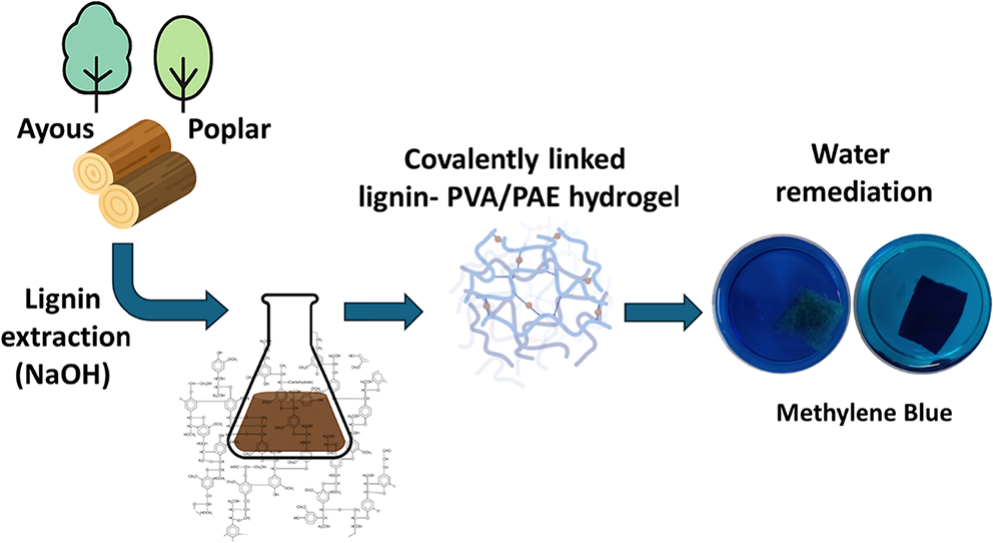
Summary of the Workflow

Overall, this study highlights the value of lignin-modified hydrogels as innovative, sustainable, and high-performance materials for the adsorption and removal of hazardous dyes, thus advancing the development of environmentally responsible solutions for wastewater purification.

## 2 Experimental Section

### 2.1 Materials

Commercial alkali lignin (a waste product from alkaline pulping, with an average molecular weight of 4337 g/mol^48^) has been purchased from Sigma-Aldrich (CAS:8068–05–1) and directly used. The other lignin samples were obtained from wood industry waste starting from Ayous and Poplar wood (Figure 1).

**Figure 1:**
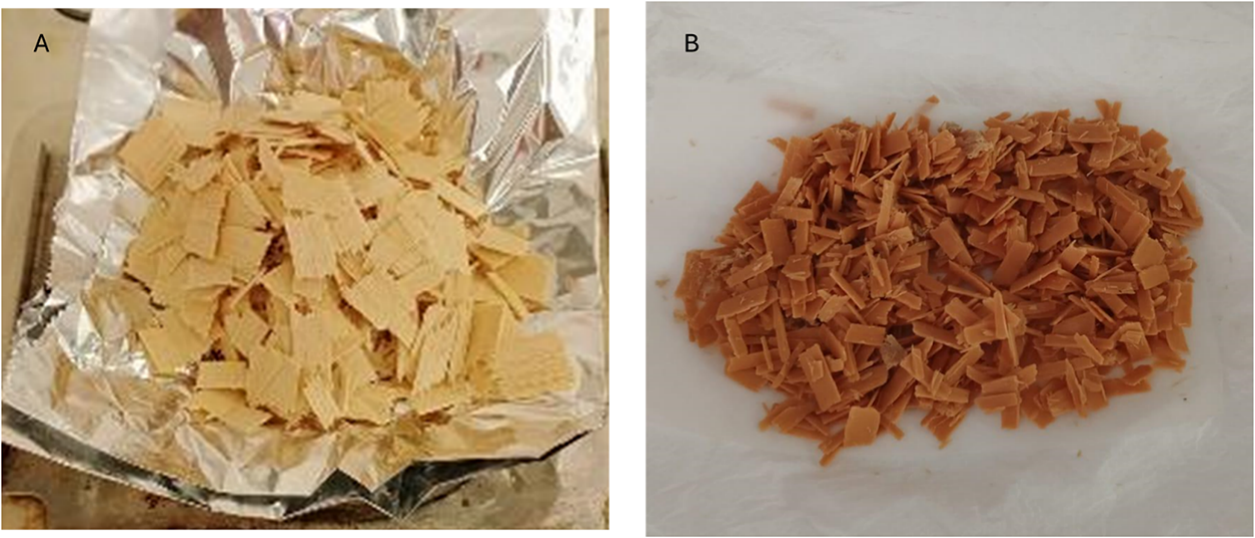
Raw materials: (A) Ayous wood and (B) Poplar wood.

Poly­(vinyl alcohol) (PVA, 99% degree of hydrolysis, 89000–97000 molecular weight), H_2_SO_4_ (95.0–98.0% w/w), and NaOH (pellets) were purchased from Aldrich, and polyaminoamide-epichlorohydrin (PAE)/water solution (solid content 12.5% w/w) was kindly provided by a supplier.

### 2.2 Lignin Extraction

The lignin extraction was performed by properly modifying a procedure reported in the literature.^49^ Chopped wooden pieces (4.5 g) were soaked in NaOH aqueous solutions (1:10 weight ratio) previously prepared at different concentrations (5, 7.5, or 10% m/V) for 3 h at 100 °C under reflux. After cooling, the mixture was filtered using a Buchner funnel, and the pH of the filtrate was adjusted from 12.6 to 2.6 by adding concentrated H_2_SO_4_ (96–98%) until lignin precipitation occurred. The mixture was then centrifuged at 6000 rpm for 20 min, and the solid residue was separated and collected. The solid residue was washed with HCl solution (pH 2) and distilled water and then dried in an oven at 50 °C for 24 h.

### 2.3 Lignin/PVA Hydrogel Preparation

#### 2.3.1 Hydro

This hydrogel was prepared with only Lig_Com_. 1.1 g of PVA was dissolved in 10.0 mL of distilled water in a flask under stirring at 80 °C (Solution A). Then, 0.5 g of lignin was dissolved in 5.0 mL of a 2% NaOH solution at 80 °C under stirring (Solution B). Solutions A and B were mixed at 80 °C under stirring until homogenization and then sonicated for 5 min. Finally, the solution was transferred to a Petri dish (9.0 cm diameter) to start the freeze–thaw cycles (3 F–T cycles).^42^ The final concentrations of lignin and PVA were 3.3 and 7.3%, respectively. The hydrogel was labeled as HydroLig_Com‑without PAE_.

#### 2.3.2 PAE-Hydro

Alternatively, a covalently linked lignin-based hydrogel was prepared by using polyaminoamide-epichlorohydrin (PAE). PVA (1.1 g) was dissolved in 10 mL of distilled water at 80–90 °C (Solution A). 0.5 g of lignin was dissolved in 5.0 mL of a 2% NaOH solution, and 0.25 g of PAE (50% w/w of lignin) was added under vigorous stirring at 80 °C for 1 h (Solution B). Solutions A and B were mixed under stirring at 80 °C for 90 min until complete homogenization. The final concentrations (m/V) of lignin, PAE, and PVA were 3.3, 1.7, and 7.3%, respectively. Finally, the solution was poured into a Petri dish (9.0 cm diameter) and subjected to freeze–thaw cycles (3 F–T cycles). The obtained materials were labeled as PVA/PAE-Hy, HyLig_Com_, HyLig_Ayo_, and HyLig_Pop._ The images of the final materials are shown in Figure S1.

### 2.4 Lignin Release Tests

To evaluate the effect of PAE on the lignin leaching, samples HyLig_com‑without PAE_ and HyLig_com_ containing the same amount of Lig_Com_ were dipped into 10 mL of distilled water for 3 h. The water was changed every 30 min until no more color change was observed. All of the washing waters were collected and mixed. The total amount of lignin released was obtained by comparing the UV–vis spectra of the collected mixed water to the lignin calibration curves (Table 1 and Figure S2). The lignin calibration curves were drawn from a 50 mg/mL stock solution (w/V) using concentrations of 0.25, 0.5, 1.0, and 2.0 mg/mL (λ_max_ = 360 nm). The amount of lignin retained within the hydrogels was determined by subtracting the amount of lignin released from the initial amount used. Lignin release test was conducted even on HyLig_Pop_ and HyLig_Ayo_ containing PAE (Table 1). All of the lignin/PVA hydrogels were subjected to this treatment before their use.

**Table 1:** Lignin Content of Hydrogels (Thickness 0.2 cm)

hydrogel	PAE	lignin remaining (%)	hydrogel area (cm^2^)
HydroLig_Com‑without PAE_	no	57	44.2
PVA/PAE-Hy	yes		44.0
HyLig_Com_	yes	72	43.0
HyLig_Pop_	yes	55	50.3
HyLig_Ayo_	yes	75	50.2

### 2.5 Characterization

The extracted lignin was characterized by X-ray diffractometry (XRD) in reflection mode on dried powders using a Philips X’Celerator diffractometer equipped with a graphite monochromator. The 2θ range was from 5 to 50° with a step size of 0.05° and a time per step of 2 s. Cu Kα radiation (40 mA, 40 kV, 1.54 Å) was used. The ζ-potential of a 3% lignin aqueous suspension was measured using a Malvern Instruments Ltd. instrument at 25 °C at the spontaneous pH.

All hydrogels prepared were lyophilized before being characterized. Lignin and hydrogel compositions were evaluated by using an FTIR spectrometer (Spectrum Two; PerkinElmer, Waltham, MA, USA) equipped with an attenuated total reflectance (ATR) module. To investigate the hydrogels’ morphology, SEM images were acquired on Au sputter-coated samples by using a Zeiss Leo 1530 Field Emission Scanning Electron Microscope. Thermal stability was evaluated by thermogravimetric analysis with a NETZSCH TG 209F1 Libra thermogravimetric analyzer in an inert atmosphere under N_2_ with a heating ramp from 25 to 600 °C and a heating rate of 20 °C/min.

To estimate the degree of swelling, the dried samples were immersed in distilled water at 25 °C for 72 h to reach swelling equilibrium.^42^ The samples were removed from the bath; the excess of unabsorbed water was quickly removed with filter paper before being weighed on an analytical balance. The swelling ratio (Sw%) was calculated according to the following formula

1 $$\mathrm{Sw}\%=\frac{W_{s}-W_{d}}{W_{s}}\cdot 100$$

where *W*_s_ is the weight of the swollen gel (g) and *W*_d_ is the weight of the dry gel (g).

Mechanical properties of the swollen hydrogels were measured using an Instron 4465 dynamometer 4465 equipped with a 100 N load cell. Stress–strain curves were recorded at a cross-head speed of 5 mm/min on strip-shaped samples (20–30 mm long, 5.0 mm wide). The thickness was measured for each sample before the test. Young’s modulus (*E*), the stress at break (σ_b_), and the strain at break (ε_b_) were evaluated. At least 5 samples were tested for each composition.

### 2.6 Dye Removal Experiments

A volume of hydrogel containing 20 mg of lignin was used for methylene blue adsorption. The experiments were conducted for 24 h at 25 °C, under stirring, into 25 mL of methylene blue dye solution (50 mg/L), adjusted to a fixed 6.5 pH.

The residual dye concentration after 24 h was determined using a calibration curve obtained by diluting 50 mg/L solution to concentrations ranging from 1.0 to 6.0 mg/L. The maximum value of absorbance at λ = 664.8 nm was used (Figure S3) to compare the residual dye concentrations. All UV–vis measurements were carried out with a PerkinElmer UV–vis–NIR Spectrometer, Lambda 19. Efficiency in dye removal (h) was calculated by eq 2.^50,51^

2 $$h=dye\;removal\;\left(\%\right)=\frac{C_{0}-C_{f}}{C_{0}}\times 100$$

where *C*_0_ is the initial concentration of dye (mg/L) and *C*_f_ is the concentration of dye in the supernatant after a fixed time (i.e., 24 h) (mg/L).

The kinetic phenomena were investigated by employing three different semiempirical adsorption kinetics: pseudo-first-order, pseudo-second-order, and intraparticle diffusion models, based on the equilibrium absorption capacity (*Q*_e_) calculated by the following equation

3 $$Q_{e}=\frac{\left(C_{0}-C_{f}\right)}{m}\times V$$

where *C*_0_ (mg/L) is the initial concentration of dye, *C*_f_ (mg/L) is the concentration of dye in the supernatant after 24 h, *V* (L) is the volume of the initial dye solution, and *m* (g) is the dry weight of the hydrogel sample.

Pseudo-first-order model for heterogeneous solid–liquid systems is formulated as follows:

4 $$\ln\left(Q_{e}-Q_{t}\right)=\ln Q_{e}-k_{1}t$$

where *Q*_e_ and *Q*_t_ (mg/g) are the amounts of dye adsorbed at equilibrium and at a given time *t* (min), respectively, using as initial conditions *Q*_t_ = 0 at *t* = 0. The slope value obtained by plotting ln­(*Q*_e_ – *Q*_t_) versus time (min) allows estimation of the constant rate *k*_1_ (min^–1^). Besides eq 3, the pseudo-second-order model allows the calculation of the reaction rate *k*_2_ (g mg^–1^min^–1^), given by the following equation

5 $$\frac{t}{Q_{t}}=\frac{1}{Q_{\mathrm{et}}}+\frac{1}{k_{2}Q_{e}^{2}}$$

By plotting the experimental data *t*/*Q*_t_ versus *t*, *Q*_e_ and *k*_2_ were assessed from both the slope and the intercept, respectively. The intraparticle diffusion model is described with the following equation

6 $$Q_{t}=k_{1}t^{1/2}+C$$

where *k_i_* is the intraparticle diffusion rate constant (mg g^–1^h^–0.5^).^50^

### 2.7 Reusability

The desorption process was conducted in a 96% ethanol solution for 2 h. After each desorption process, the hydrogel was quickly washed with distilled water before the subsequent adsorption process. The regenerated hydrogel underwent ten additional adsorption/desorption cycles. The reuse efficiency of the hydrogel was evaluated by using the following equation

7 $$Re_{n}\left(\%\right)=\frac{q_{0}}{q_{n}}\times 100$$

where *n* is the number of adsorption/desorption cycles, and Re*_n_* is the reusing efficiency after *n* adsorption/desorption cycles. *q_n_* is the adsorption capacity after *n* adsorption/desorption cycles, and q_0_ is the initial adsorption capacity of the hydrogels.

## 3 Results and Discussion

### 3.1 Lignin Extraction, Yield, and Characterization

Two lignins, deriving from the Poplar (Populus L., Lig_Pop_) and Ayous (Triplochiton scleroxylon, Lig_Ayo_), not treated wood residues obtained during the industrial process of Alpi Spa, were extracted with NaOH and characterized. A schematic representation of the lignin extraction process is reported in Figure 2. To optimize the process, three different concentrations of NaOH have been employed: 5, 7.5, and 10% w/V; the results are shown in Table S1 for Ayous wood, as an example. The maximum yield was obtained using a 10% solution of NaOH, which was used for the extraction of lignin from Poplar and even Ayous wood. The process, repeated in triplicate, showed that the yield was 11.0 ± 0.7% for the Poplar wood and 3.6 ± 0.8% for the Ayous wood. The data agree with those expected from the two different woods.^52^

**Figure 2:**
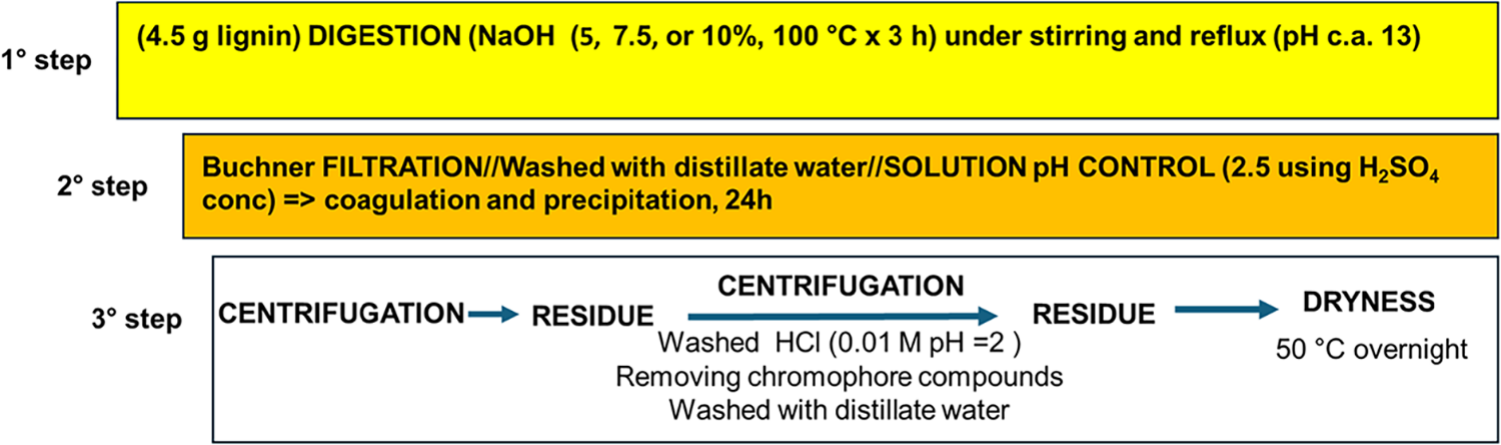
Wood lignin extraction procedure.

The optical and SEM images of extracted lignin (Lig_Ayo_ and Lig_Pop_) and commercial lignin (Lig_Com_) are reported in Figure 3. Commercial alkaline lignin appears as spherical agglomerates with a smooth surface (Figures 3b and S4). In contrast, lignin derived from raw wood materials has a rougher and more uneven surface, particularly in the case of Lig_Ayo_.

**Figure 3:**
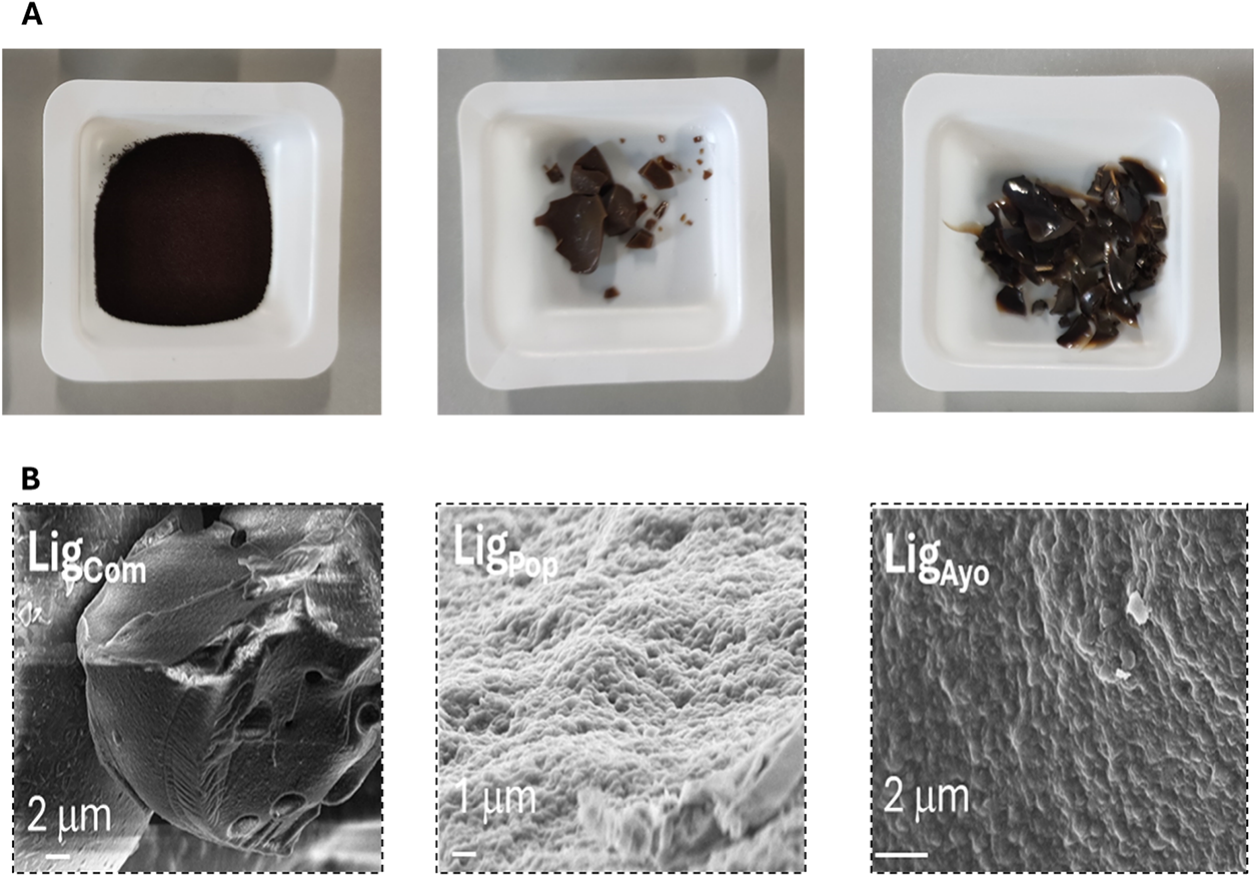
Optical (A) and SEM images (B) of commercial (Lig_Com_), Poplar wood (Lig_Pop_), and Ayous wood (Lig_Ayo_) alkali lignin.

Water suspensions obtained by pouring 1.5 mg of solid in 50 mL of distilled water were prepared to evaluate the spontaneous lignin’s pH and zeta potential. The pH measurements were performed under stirring at 25 °C by using an AMEL pH meter. The ζ-potential analysis of the lignin suspensions gives an overview of both the stability of the particles in the medium and the repulsive or attractive electrostatic forces between them. The data are reported in Table 2.

**Table 2:** Spontaneous pH and Z Potential under Stirring

sample	pH	Z potential (mV)	standard deviation (mV)	Z deviation (mV)	conductivity (mS/cm)
Lig_Com_	7.1	–18.4	4.5	9.6	0.024
Lig_Ayo_	5.0	–11.7	3.6	3.6	0.090
Lig_Pop_	5.2	–25.7	5.7	12.7	0.045

The mean zeta potential values were between −11.7 and −26.0 mV, indicating repulsive interactions that reduce the probability of agglomeration and contribute to greater stability. Moreover, the negative Z potential value observed for all of the lignin may be due to the presence of negatively charged ionic groups such as hydroxyls, carboxyls, and phenolics on the surface of lignin,^53^ and a remarkable difference has been observed between lignin extracted from Ayous and Poplar.

FTIR spectroscopy is an important tool for structural characterization of hardwood and softwood samples: in fact, softwood mainly contains only guaiacyl (G) units, while hardwood contains both guaiacyl (G) and syringyl (S) units, and the syringyl ratio (syringyl/(syringyl + guaiacyl)) varies among species.^47^

All samples were characterized by a broad O–H band at 3400 cm^–1^, C–H stretching of methyl or methylene groups around 2900 cm^–1^, aromatic skeletal vibrations in the range of 1605–1590 cm^–1^, C–C stretching of aromatic rings around 1510 cm^–1^, C–H stretching of aromatic rings around 1460 cm^–1^, and CH vibration of methyl group at 1420 cm^–1^. In addition, there are some peaks peculiar to soft and hardwood lignins, as reported in the literature (Figure 4). The peak at 1030–1035 cm^–1^ is allocated to aromatic C–H in-plane deformation when the amount of G units is higher than S units. This region is also allocated to C–O deformation in primary alcohols and C=O stretch (unconjugated). In contrast, aromatic C–H in-plane deformation (typical for S units) plus secondary alcohols plus C=O stretch are allocated in the range of 1120–1130 cm^‑1^.^54^

**Figure 4:**
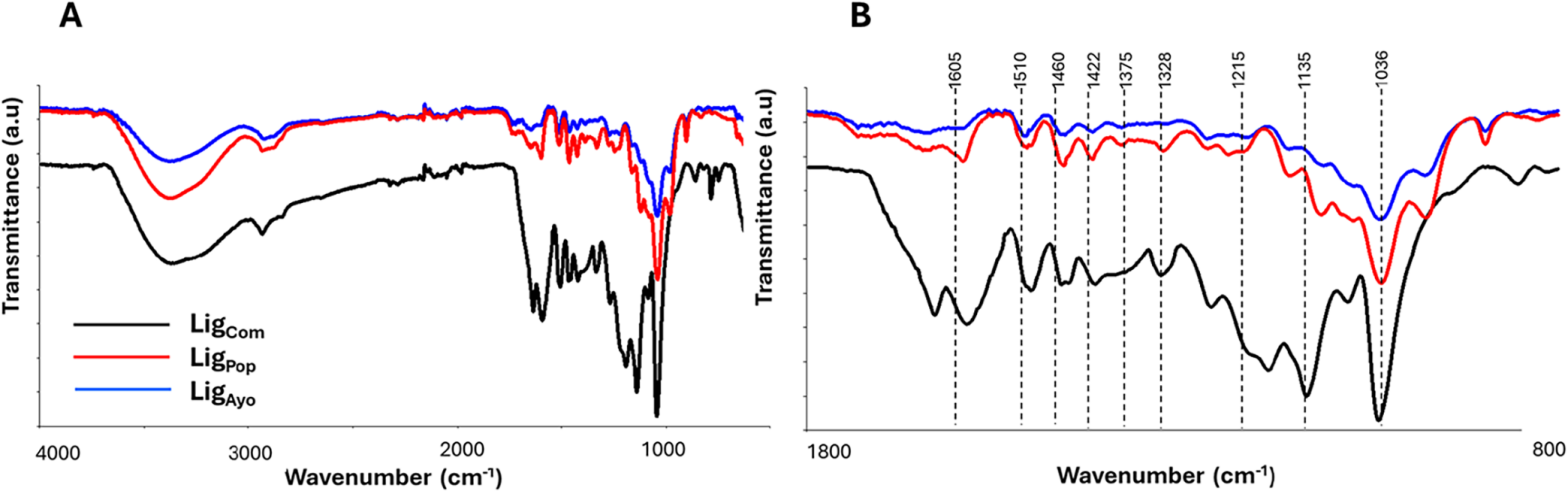
(A) ATR-FTIR spectra and (B) enlargement of spectra in absorbance from 1800 to 800 cm^–1^.

Peaks in the wavenumber range of 1266–1270 cm^–1^ are associated with G ring vibrations, and they are not present in hardwood samples. Furthermore, the peak in the range of 1515–1505 cm^–1^ is attributed to aromatic skeletal vibrations when G units are higher than S units. Its comparison with the band in the range 1460–1470, due to C–H in-plane deformation (typical for S units), gives information about the soft or hard nature of wood. The spectra confirmed that Ayous has the characteristic of softwood (1510 > 1460) while Poplar can be mainly described as hardwood (1510 < 1460).^55–58^

The diffractogram of commercial lignin (Lig_com_) showed a broad signal centered around 20°/2 theta, revealing an amorphous structure (Figure S5). Patterns obtained from Lig_Ayo_ and Lig_Pop_ showed an amorphous structure too, where the main reflections, centered at 19 and 21°, respectively, align with the values reported in the literature^59^ for soft and hardwood, respectively.

### 3.2 Lignin/PVA Hydrogel

Physically cross-linked lignin/PVA hydrogels were obtained via a freezing–thawing cycle gelation process and using water as solvent, without adding any gelation-inducing agent. Increasing the structural stability of hydrogel adsorption materials is crucial to preserving their adsorption efficiency and durability in water treatment applications. For this purpose, epichlorohydrin (ECH), which forms covalent bonds with the hydroxyl groups of PVA under basic conditions, is widely used as a cross-linking agent to increase the structural stability of PVA-based hydrogel.^40,60,61^ Despite its chemical effectiveness, the significant health risk of ethylene has prompted the search for safer alternatives. Among them, polyamideamine epichlorohydrin (PAE), a cationic water-soluble thermosetting functionalized with an azetidine group that can covalently react with the carboxylic groups, represented a suitable substitute, and it is widely used in the papermaking industry to impart wet strength to paper products.^41,50,51^ For this reason, PAE was integrated into the PVA hydrogel preparation to enhance lignin stability via additional chemical cross-linking reactions (Scheme 2).

**Scheme 2:**
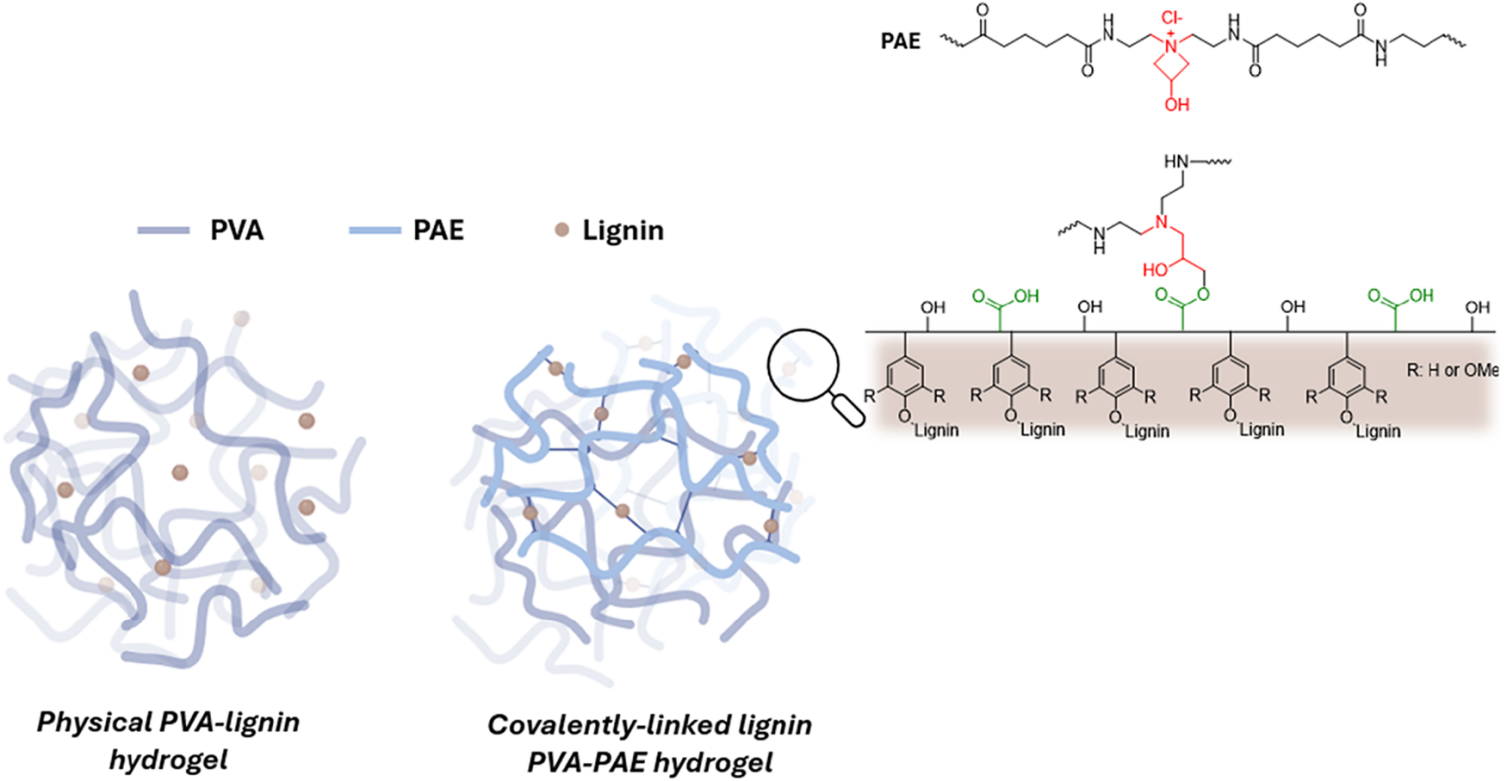
Schematic Representation and Mechanism Involved in the Preparation of Covalently Linked PVA/PAE Hydrogels

The effect of the cross-linking process on the structural stability of the hydrogels and lignin release was evaluated at a fixed pH of 6.5 using commercial lignin (Table 1). By comparing the data, a lignin leakage of 43% was obtained from HydroLig_Com_-_without PAE_, whereas only 28% leakage was observed for HyLig_Com_ prepared with 1.7% (m/V) PAE addition.

Following these results, the hydrogels were cross-linked in the presence of 1.7% (m/V) PAE; the amount of lignin remaining in the hydrogels after treatment is reported in Table 1. The freeze-dried PVA/PAE-Hy, as a reference, and the covalently linked lignin-based hydrogel’s morphology were investigated by scanning electron microscopy images and are reported in Figure 5. The longitudinal sections of the hydrogel-containing lignin (Figure 5B–E) revealed a highly porous three-dimensional network structure with ultrathin walls, whereas a more compact and smoother surface is exhibited by PVA/PAE-Hy (Figure 5A).

**Figure 5:**
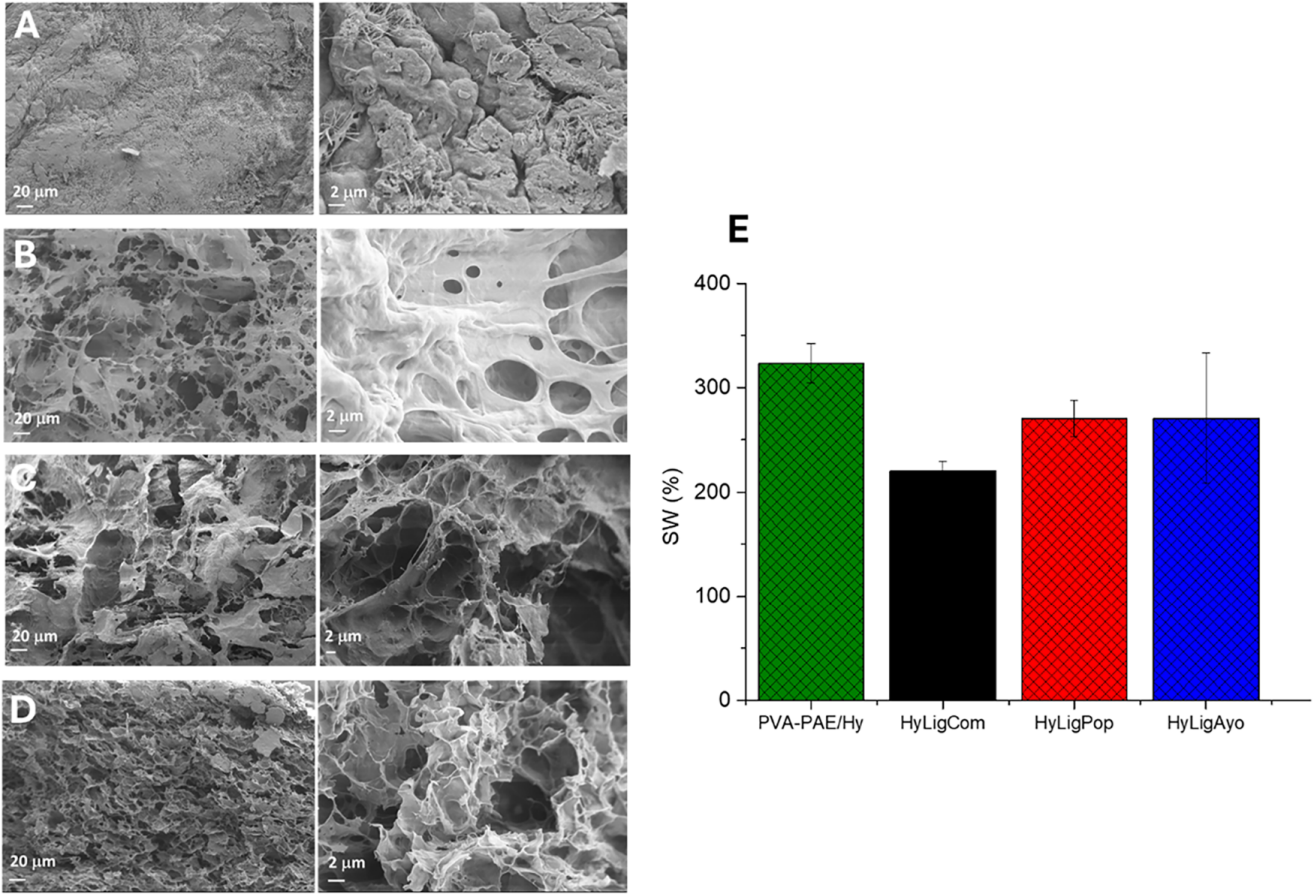
SEM images of (A) PVA/PAE-Hy, (B) HyLig_Com_, (C) HyLig_Ayo_, and (D) HyLig_Pop._ All of the images are reported at two magnifications: 620 x and 5.5 Kx. (E) Swelling ratios (%) of the PVA/PAE-Hy and HyLig hydrogels at pH 6.5.

During hydrogel swelling, water first diffuses into the polymer network, followed by extension of polymer chains. Adding 3.3% (m/V) lignin results in more hydroxyl groups in PVA being consumed to form more cross-linking points or physical interactions that restrict the hydrogel network’s ability to swell extensively in water and reduce hydrophilicity and water uptake (Figure 5E).^64,65^ Additionally, lignin’s high hydrophobicity can limit the diffusion of water molecules into the hydrogel, leading to a lower equilibrium swelling ratio.^65^ The differences observed with various lignins can be attributed to a difference in average molecular weight and content of phenolic hydroxyl groups of the lignin.^60^

The infrared spectra of PVA/PAE-Hy and HyLig are shown in Figure 6A. From the literature,^62,63^ PAE exhibited the characteristic absorption bands at approximately 1151 and 1051 cm^–1^, which can be attributed to the bending vibration of quaternary amino groups (C–N+) and C–OH groups belonging to azacyclic butanol.

**Figure 6:**
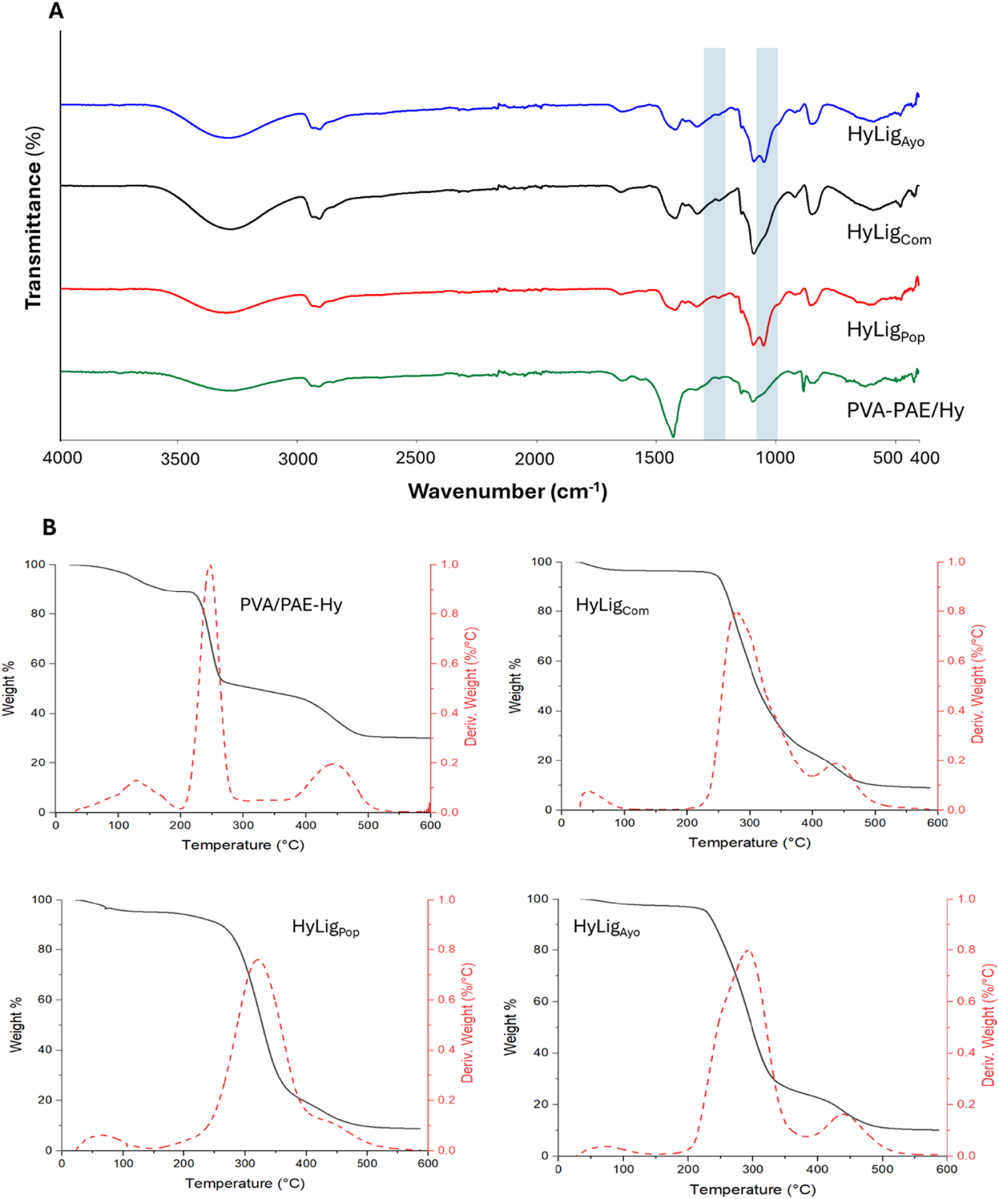
(A) ATR-FTIR spectra of the PVA/PAE-Hy and HyLig_s_ hydrogels (highlighted area indicates lignin peaks). (B) Thermogravimetric analyses of PVA/PAE-Hy and HyLig_s_ hydrogels.

The strong absorption peaks at 3332 and 919 cm^–1^ of the PVA hydrogel were attributed to the O–H stretching vibration and the bending vibration of CH_2_.^66^ The HyLig_s_ showed characteristic absorption bands at 1605, 1375, and 1036 cm^–1^ of the lignin.

The thermal stability of the adsorbent plays a crucial role in evaluating its performance in wastewater treatment applications. Figure 6B depicts the TGA and DTG curves of the PVA–PAE/Hy and HyLig_s_ samples. Thermal degradation can be divided into three major stages. The first stage ranges from room temperature to approximately 100 °C; the small weight loss in this stage has been attributed to the evaporation of water absorbed in the hydrogels. The second stage, from 180 to 400 °C, was the major decomposition period, during which the samples were broken down into small molecules and gaseous products.^67^ The last stage is above 410 °C, in which the residues were further degraded into gas and carbonaceous residue. The residual weight percentages of PVA/PAE-Hy and HyLig_s_ are 30, and 10%, respectively. The addition of 3% lignin in the hydrogels increases the thermal degradation onset temperature (*T*_o_) from 200 °C for PVA/PAE-Hy to 210, 216, and 234 °C for HyLig_Ayo_, HyLig_Pop_, and HyLig_Com_, respectively. Similarly, the maximum weight loss temperature (Tmax) increases from 250 °C for PVA/PAE-Hy to 283, 292, and 325 °C for HyLig_Com_, HyLig_Ayo_, and HyLig_Pop_, respectively. This indicates that HyLig_s_ presents heat-resistant properties.^65^

### 3.3 Dye Adsorption Studies

Methylene blue (MB) adsorption experiments were conducted using HyLig hydrogels in 25 mL of a 50 mg/L MB solution with stirring at 25 °C. The changes of the UV–vis spectra were recorded after 24 h. The amount of hydrogel was set to contain the equivalent of 20 mg of lignin (HyLig_Com_, HyLig_Ay_, HyLig_Pop_) (see details in Table 3).

**Table 3:** Removal or Decolorization Efficiency and Equilibrium Adsorption Capacity for HyLig Hydrogels

hydrogel	removal efficiency *h* in 24 h (%)	equilibrium adsorption capacity *Q*_e_ (mg/cm^3^)	equilibrium adsorption capacity *Q*_e_ (mg/g_lig_)^b^	hydrogel volume used (cm^3^)
HyLig_Com_	64	1.6	40	0.50^a^
HyLig_Pop_	60	1.0	38	0.73^a^
HyLig_Ayo_	88	2.1	54	0.54^a^
PVA/PAE-Hy	9	0.2		0.60

a Volume containing c.a. 20 mg of Lignin.

b *Q*_e_ calculated vs the amount of lignin contained in the hydrogel (g_lig_).

A fixed pH of 6.5 (adjusting with a small amount of NaOH or HCl) was used for all of the experiments.^68^ At pH 6.5, alkali lignin is likely to be negatively charged due to the presence of ionizable groups such as phenolic and carboxylic groups.^69^ These groups can donate or accept protons, depending on the pH, influencing the overall charge of the lignin molecule. At a pH below the typical p*K*_a_ of phenolic groups (around 10), these groups are predominantly in their protonated, neutral form. However, if a phenolic group has a p*K*_a_ lower than 6.5, it may still be deprotonated at this pH. With regard to the carboxylic groups, they have a lower p*K*_a_ (around 4–5) and are likely to be partially or fully deprotonated at pH 6.5, contributing an overall negative charge. At the same pH value, MB is present as a single species, exhibiting a positive charge (Figure S6). The removal efficiency *h* and the estimated equilibrium adsorption capacity *Q*_e_ are reported in Table 3. *Q*_e_ was calculated concerning the volume of the hydrogel used (mg/cm^–3^) or the amount of lignin present in the volume of the hydrogel used (mg/g_lig_). Compared with PVA/PAE-Hy, the presence of lignin in the hydrogel provides a significant increase in removal efficiency (*h*) and equilibrium adsorption capacity (*Q*_e_) for all of the samples.

As reported in to the literature,^70^ multiple interactions are present in the adsorption of MB on lignin, mainly hydrogen bonds between the aliphatic and heterocyclic N atoms of MB and the aliphatic and benzylic hydroxyls of lignin; then electrostatic attraction and van der Waals interactions between the positively charged nitrogen of MB and the negatively charged carboxylate and phenolate groups of lignin and finally π–π interactions between the aromatic rings of MB and lignin are involved. In any case, other factors, such as cooperative effects, can significantly influence adsorption.

The highest Qe obtained for HyLig_Ayo_ relative to HyLig_Com_ can be justified by considering the lower swelling capability of the latter. In contrast, the lower removal efficiency observed for HyLig_Pop_ can be justified by the greater water repellence characteristic of poplar lignin. Moreover, the lower z potential of HyLig_Ayo_ suggests that a mechanism other than the electrostatic attraction between the negative lignin and the positive MB dye became more dominant. Due to the higher content of G units in the Lig_Ayo_, it is conceivable a π–π* stacking.

The adsorption capacity of HyLig_Ayo_ toward MB falls within the range of adsorption capacity (from 1047.7 mg/g with a bentonite-doped lignin hydrogel sphere to 43.0 mg/g with lignin derivative magnetic hydrogel microspheres)^71^ reported for the previous lignin composite hydrogels (Table S2).^71^

Adsorption kinetic studies were conducted through the results of changes in the MB adsorption amount over time at a fixed starting concentration of 50 mg/L (Figure S7). The effect of contact time on the adsorption amount of MB on HyLig is displayed in Figure 7A. It can be observed that the adsorption amount increases sharply in the first 20 min, and then the adsorption rate is remarkably slowed down with HyLig_Pop_ and HyLig_Com_, and a linear increase until 120 min is obtained with HyLig_Ayo_ and PVA/PAE-Hy. The results of adsorption over processing time were analyzed using the pseudo-first- and pseudo-second-order models, which are representative kinetic models that can classify physicochemical adsorption phenomena. The linear fit results are reported in Figures 7B and S8, and Table S3. At the investigated dye concentration, HyLig_Com_ and HyLig_Pop_ showed a higher *R* ^2^ value in the pseudo-second-order model than in the pseudo-first-order model for MB adsorption, which means that MB adsorption occurs through chemical processes. This is consistent with the results obtained with polymer-based adsorbents, especially those in which electrostatic attraction is the main driving force,^40^ and in accordance with the best fit (highest *R* ^2^ values) observed with the pseudo-second-order model exhibited by the pristine lignin (Figure S9 and Table S4). On the contrary, HyLig_Ayo_ presents a higher *R* ^2^ value in the pseudo-first-order model, indicating that the adsorption kinetic is fast and is mainly controlled by physisorption.^72^ This behavior is consistent with a guaiacyl (G)-rich lignin cross-linked in a PVA/PAE hydrogel in which the electrostatic interaction with MB is attenuated by a more negative surface.

**Figure 7:**
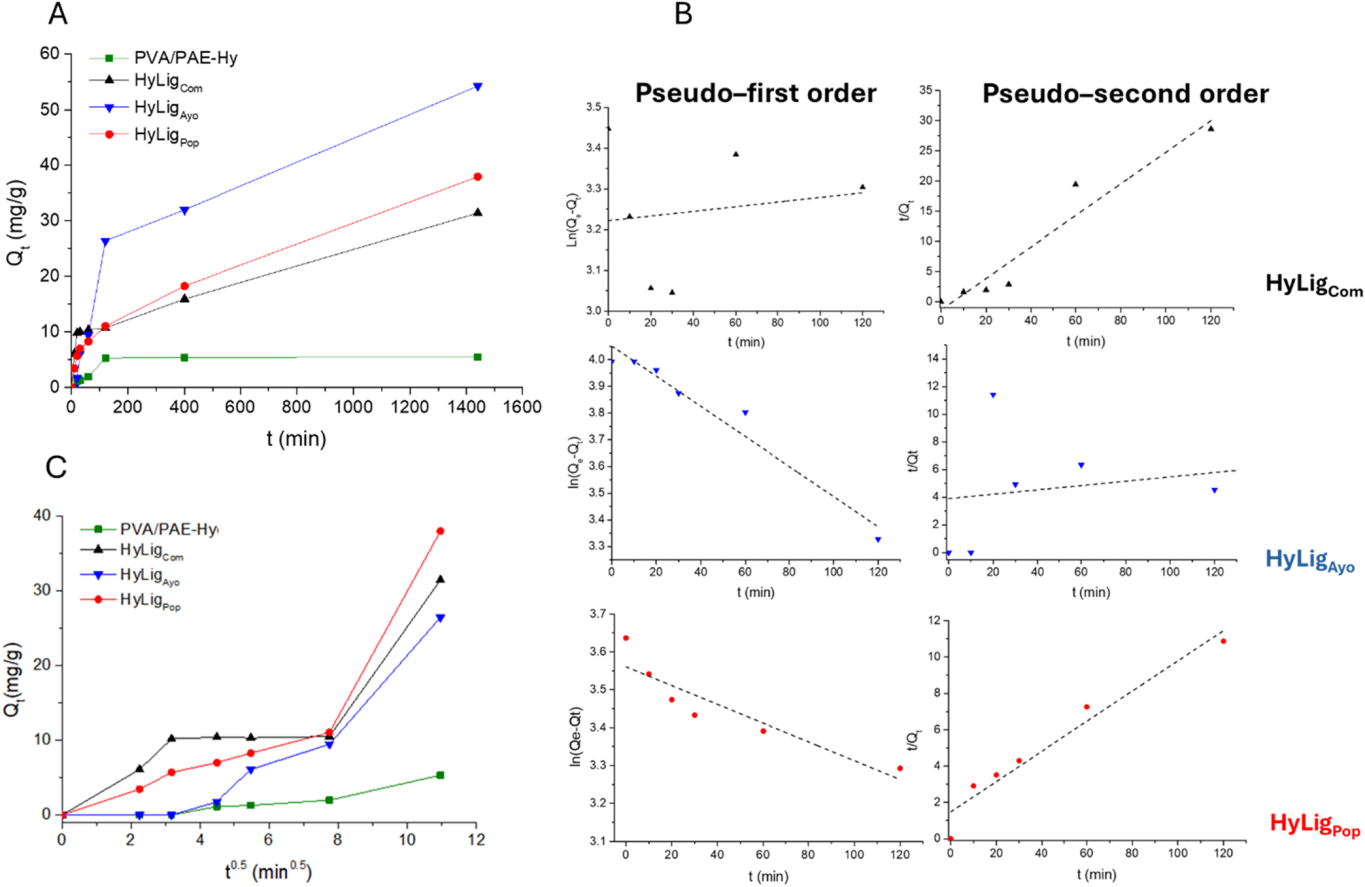
Kinetic studies at 25 °C with different HyLig: (A) effect of the contact time on the MB removal, (B) pseudo-first- and pseudo-second-order models, and (C) intraparticle diffusion model for HyLig adsorbent.

In the interparticle diffusion model shown in Figure 7C, MB removal occurs in three steps. The initial rapid MB removal within the first 20 min is mainly attributed to the rapid adsorption of MB onto the lignin microparticles exposed on the hydrogel surface. Meanwhile, once this surface adsorption reaches saturation, MB molecules in the wastewater begin to diffuse into hydrogel’s internal structure, causing a slowdown in the adsorption rate.^40^ This is then followed by another phase of rapid adsorption driven by lignin present inside the hydrogel structure. The difference in adsorption rate observed with the different lignins may be related to the complex adsorption mechanism, which involves (i) electrostatic attractions, (ii) van der Waals forces, (iii) the formation of active hydrogen bonds between the phenolic hydroxyl group of lignin and MB molecules, and (iv) strong π–π* stacking interactions.^40^ Table 4 reports the calculated kinetic parameters. For comparison, Table S5 shows the kinetic data reported in the literature for similar adsorbents.

**Table 4:** Calculated Parameters for Kinetic Models and Intraparticle Diffusion Models of MB on HyLig

model	parameters	PVA–PAE/Hy*	HyLyg_Com_*	HyLyg_Ayo_*	HyLyg_Pop_*
Pseudo-1^st^ order	*k*_1_ (min^–1^)	11.42 × 10^–2^	0.13 × 10^–2^	1.30 × 10^–2^	0.46 × 10^–2^
	*q*_cal_ (mg/g^–^)	12	25.07	57.48	35.16
	*R* ^2^	0.9155	0.1515	0.9780	0.9173
Pseudo-2^nd^ order	*k*_2_ (g/(mg^–^min))	3.92 × 10^–3^	0.071 × 10^–3^	0.063 × 10^–3^	2.67 × 10^–3^
	*q*_cal_ (mg/g)	5.50	37.97	63.53	13.51
	*R* ^2^	0.9957	0.9564	0.9092	0.974
intraparticle diffusion	*K*_dI_ (g/(mg^–^min^1/2^))	0.54	2.49	2.98	1.42
	*R* ^2^	0.9396	0.9640	0.9946	0.9931

### 3.4 Regeneration Test

The reusability of HyLig hydrogels was evaluated by performing cycles of adsorption/desorption.^53^ HyLig_Ayo_ was selected for its better performance in the MB adsorption test previously described.

A proper hydrogel volume (see Table 3 for details) was soaked in 25 mL of MB solution (50 ppm) for 2 h at 25 °C, and then the amount of MB adsorbed was evaluated by UV–vis spectroscopy. After each adsorption cycle, the hydrogel was regenerated by treatment with 20 mL of ethanol (95%) for 2 h, changing the solution after 1 h. This adsorption/desorption cycle was repeated ten times, as shown in Figure 8A. The initial adsorption efficiency of 88% dropped to 73% after 3 cycles and maintained a value around 60% up to the sixth cycle. As the number of cycles increased, the desorption efficiency gradually decreased.

**Figure 8:**
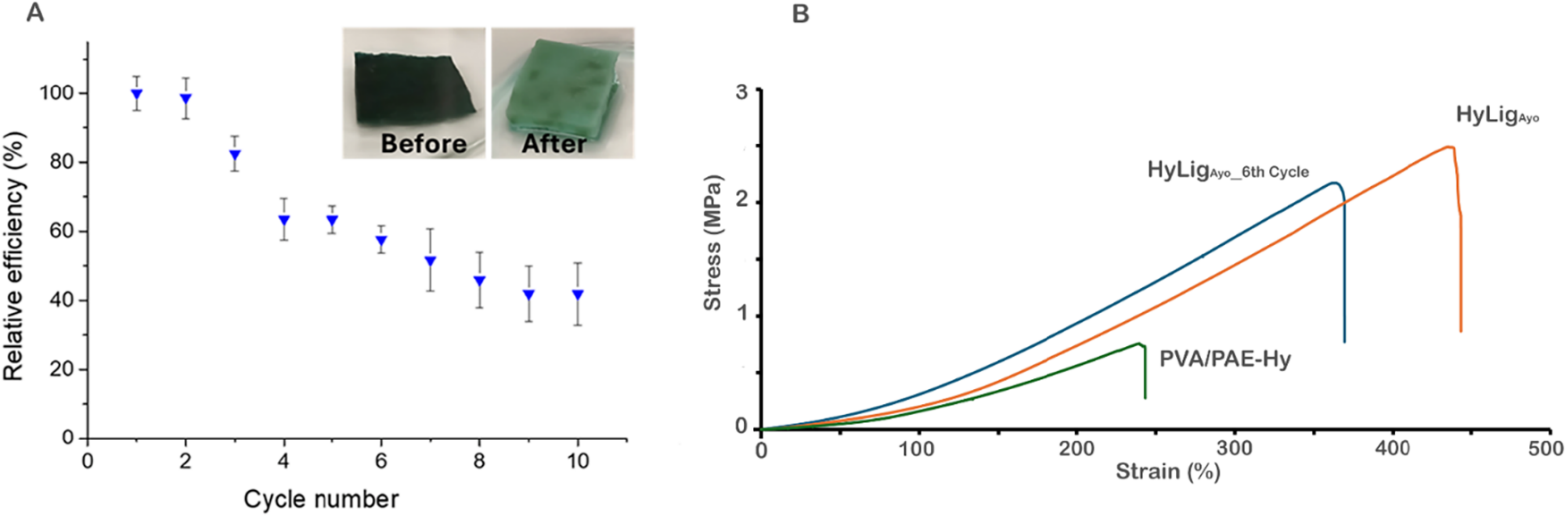
(A) Removal efficiencies of MB dye on HyLig_Ayo_ hydrogel (conditions: 25 °C, dye concentration 50 ppm, pH = 6.5, contact time: 120 min) at different consecutive cycles; (B) stress–strain curves of PVA/PAE-Hy, HyLig_Ayo_ as prepared, and HyLig_Ayo_ after six cycles of adsorption/desorption.

To investigate the material properties and the stability of the hydrogels over multiple regeneration cycles, tensile mechanical tests were performed (Figure 8B). The PVA/PAE-Hy hydrogel exhibited a maximum stress at break of 0.75 ± 0.15 MPa, a Young’s modulus of 0.43 ± 0.15 MPa, and a strain at break of 230 ± 60%. According to the literature, the addition of lignin significantly enhances the mechanical properties: specifically, the HyLig_Ayo_ hydrogel shows more than a 2-fold increase in both maximum stress (2.2 ± 0.3 MPa) and strain at break (470 ± 0.15%), while the effect on the Young’s modulus is less pronounced (0.69 ± 0.12 MPa). Notably, the hydrogel maintains its mechanical stability even after six adsorption/desorption cycles, with only an ∼20% decrease in maximum stress and an ∼30% reduction in strain at break. Moreover, the stability of Lig_Ayo_ after several cycles of MB adsorption was investigated by collecting diffraction patterns before and after 7 absorption/desorption cycles. Only a slight decrease in the intensity of the diffraction broadband attributable to lignin is observed (Figure S10).

## 4 Conclusion

The biomass residues generated during the production of wood derivatives can be effectively valorized through the green extraction protocol developed in this study. Lignin was extracted from Ayous and Poplar using sustainable methods that employ only diluted sodium hydroxide as the active chemical, achieving satisfactory yields ranging from 3.6 to 11.0%. The extracted lignin was integrated into PVA-based hydrogels prepared *via* the freezing–thawing method. Furthermore, a suitable strategy has been developed to limit the lignin leaching using a nontoxic cross-linker, polyamide epichlorohydrin (PAE), that can selectively react on the carboxylic groups of the lignin. All materials were widely characterized by spectroscopic, thermal, and microscopic analysis. The adsorption capacity of methylene blue (MB), a cationic dye commonly used in textile and paper manufacturing, was evaluated at neutral pH using the extracted lignin both as powders and in hydrogel systems (HyLig). Adsorption tests and kinetic models (pseudo-first-order, pseudo-second-order, and interparticle diffusion models) were applied to investigate the adsorption mechanism and dye removal efficiency. Kinetic studies have highlighted that Lignin extracted from Ayous exhibited superior adsorption performances compared with Poplar, probably due to the intrinsic structural differences. The presence of cross-linking significantly increased the amount of lignin retained within the hydrogel matrix, thus notably improving the adsorption efficiency matched to the bulk materials.

Hydrogels containing Ayous lignin (HyLig_Ayo_) achieved 88% removal of methylene blue within 24 h and maintained around 60% efficiency over more than six adsorption/desorption cycles. Kinetic analysis revealed that the interaction between methylene blue and lignin follows a second-order model for HyLig_Pop_ and HyLig_Com_, suggesting that chemical interactions occur between the cationic dye and the carboxylate groups of lignin. Whereas a first-order kinetics is observable for HyLig_Ayo_. The results highlight the promising potential of lignin-PVA hydrogels for the effective removal of dye contaminants from wastewater streams, allowing easy recovery of the absorbent for further cycles. This approach can be directly applied to industrial effluent treatment, especially in manufacturing sectors such as textiles and paper production, contributing to sustainable and eco-friendly wastewater purification technologies.

## Supplementary Material



## Data Availability

All data supporting the findings of this study are included within the article and the Supporting Information.

## Notes

Optical images of the hydrogels (Figure S1); UV–vis spectra and calibration curve for Lig_Com_ (Figures S2 and S3); SEM images of commercially available alkaline lignin (Figure S4); X-ray diffraction patterns of lignins (Figure S5); Lignin and methylene blue microspecies distribution at pH 6.5 (Figure S6); Adsorption spectra of MB at different times on different hydrogels and removal rate (Figure S7); Kinetic studies of PVA/PAE-Hy (Figure S8); Kinetic studies of pristine lignin (Figure S9); Diffraction patterns of Lig_Ayo_ collected before and after 7 absorption/desorption cycles (Figure S10); Yield of lignin extracted from Ayous wood with different amounts of NaOH (Table S1); The previously reported hydrogel composite for the MB adsorption (Table S2); Linear fit result for pseudo-first-order and pseudo-second-order models (Table S3); Calculated parameters for kinetic models and intraparticle diffusion models of MB on lignin powder (Table S4); Comparison of kinetic parameters for various lignin-based adsorbents (Table S5) (PDF)

## Abbreviations

PVA: poly­(vinyl alcohol)
PAE: polyamide epichlorohydrin
TGA: thermogravimetric analysis
MB: methylene blue
PVA/PAE-Hy: hydrogel obtained with PVA and PAE
HyLig: hydrogel (PVA and PAE) containing lignin (Commercial, Ayous, Populous = HyLigCom, HyLigAyo, HyLigPop)
