# Supplementary material for: Covalently Linked Poplar and Ayous Lignin-Based Hydrogels: Sustainable Materials for Water Remediation
Source: ACS Omega. 2026 Jan 27;11(5):7998–8010. doi: 10.1021/acsomega.5c10163 (PMC12903011; doi:10.1021/acsomega.5c10163)
Supplement: Supplementary file 1 [file ao5c10163_si_001.pdf]

Covalently linked Poplar and Ayous lignin-based hydrogels: sustainable materials for water remediation

Lamyea Yeasmin[a,e], Valentina Di Matteo[a], Angelica Giovagnoli[a], Stefano Scurti[a], Maria Cristina Cassani[a,b], Silvia Panzavolta[d,] Asma Munir[d], Ilaria Ragazzini[f] and Barbara Ballarin,[a,b,c*]

[a]Department of Industrial Chemistry "Toso Montanari", Bologna University, Via Piero Gobetti 85, I-40129, Bologna, Italy. *UdR INSTM of Bologna*

[b]Center for Industrial Research-Advanced Applications in Mechanical Engineering and Materials Technology CIRI MAM University of Bologna, Viale del Risorgimento 2, I-40136 Bologna, Italy.

[c]Center for Industrial Research-Fonti Rinnovabili, Ambiente, Mare e Energia CIRI FRAME University of Bologna, Viale del Risorgimento 2, I-40136 Bologna, Italy.

[d]Department of Chemistry "Giacomo Ciamician", University of Bologna, Via Piero Gobetti 83, I-40129, Bologna, Italy.

[e]Politecnico di Torino, Corso Duca degli Abruzzi, 24 – 10129 Torino, Italy

[f]ALPI, Research Manager Viale della Repubblica 34, 47015 Modigliana (FC) Italy

*To whom correspondence should be addressed. E-mail: barbara.ballarin@unibo.it (B.B), tel: +39 051 2093700.

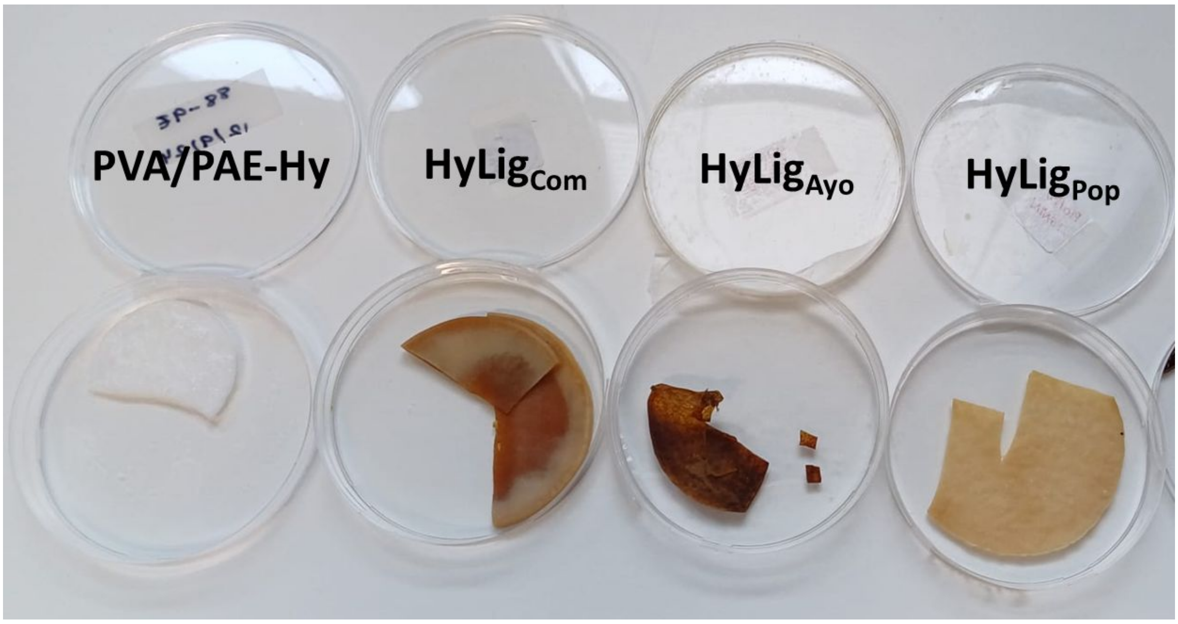

Figure S1. Optical images (from left to right) of: Pristine hydrogel and PAE-hydrogels containing the different lignin

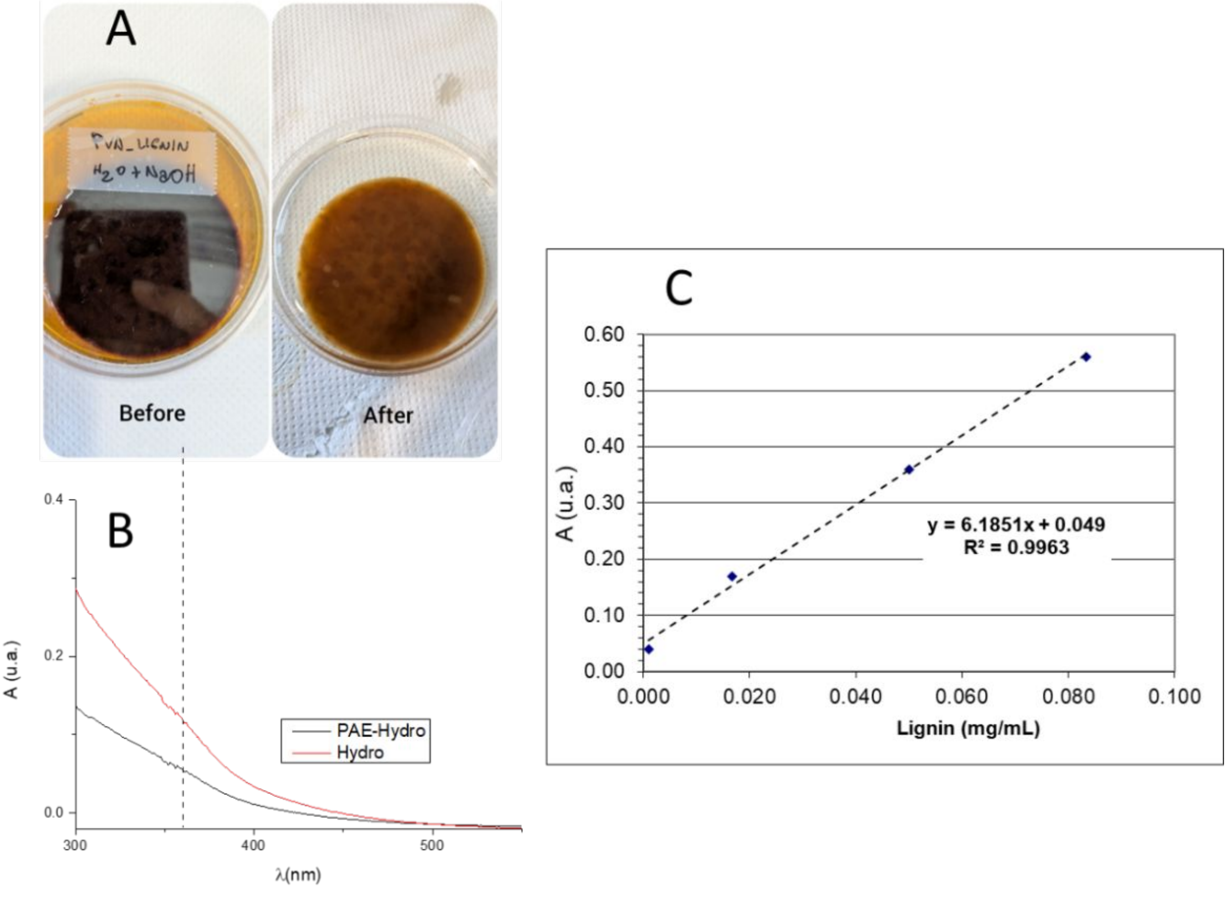

Figure S2. A) 1st vs. 10th washing cycle of hydrogel into 10 ml distilled water¸ B) UV-Vis spectra of the released Lig_{Com}, C) calibration curve for Lig_{Com}; the λmax at 360 nm was used.

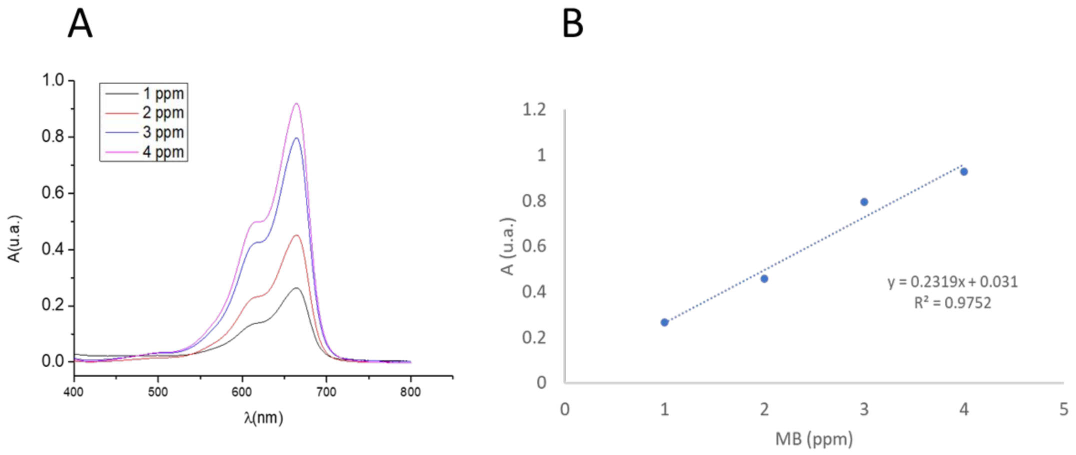

Figure S3. A) MB spectra¸ B) calibration curve, the λ_{max} at 664.8 nm was used.

Table S1. Yield of Lignin extracted from Ayous wood with different amounts of NaOH

**[NaOH] (% m/V)**	**Starting weight (g)**	**Final weight (mg)**	**Yield (%)**
5	4.5	91.0	2.0
7.5	4.5	116.0	2.6
10	4.5	162.6	3.6

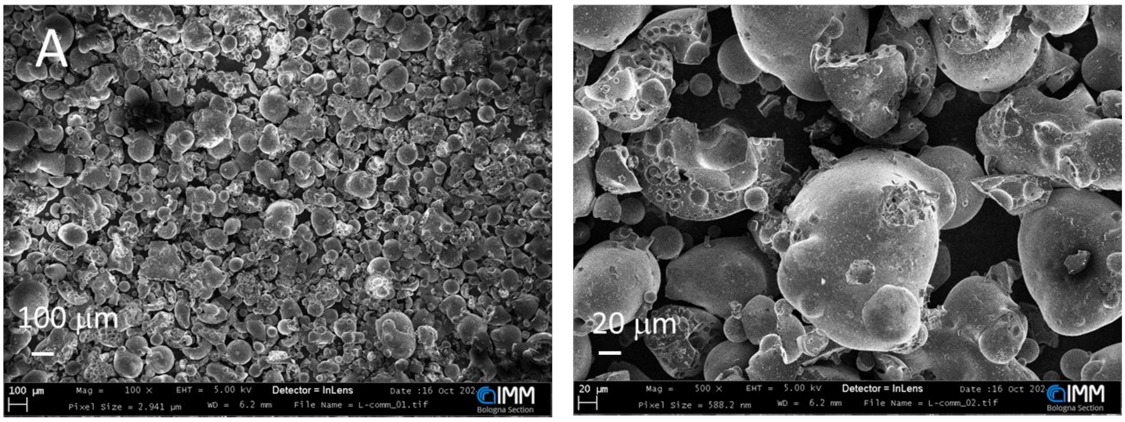

Figure S4. SEM images of commercially available alkaline lignin (Lig_{Com}) at different magnifications

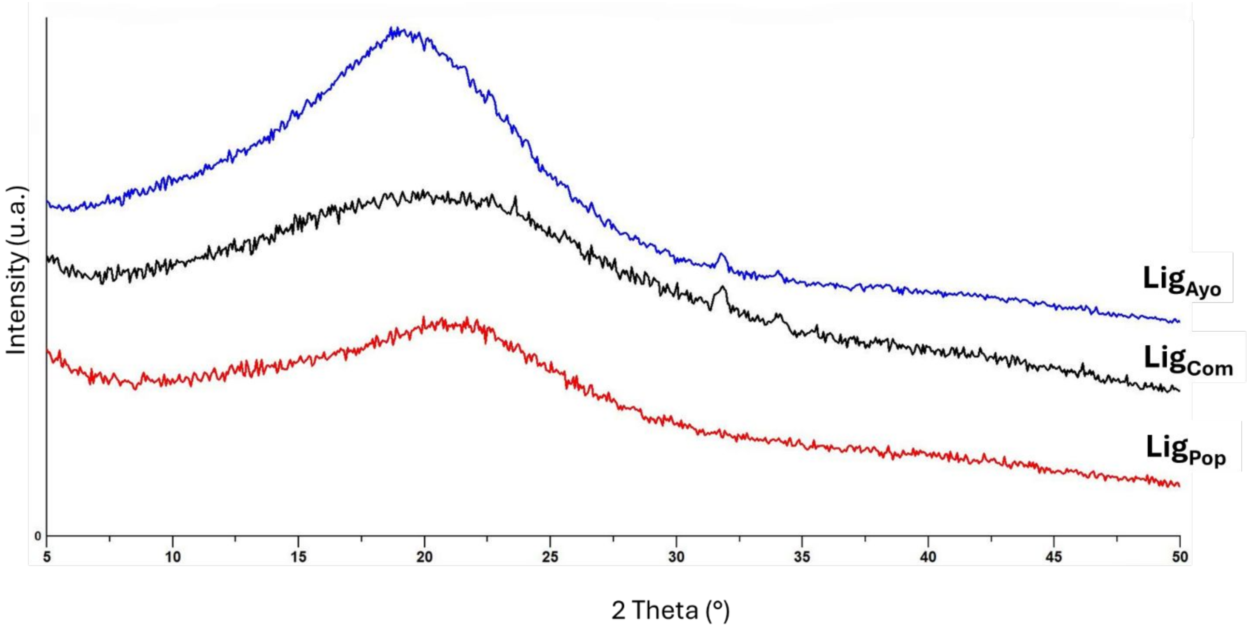

Figure S5. X-rays diffraction patterns of Lig_{Com}, Lig_{Pop} and Lig_{Ayo},

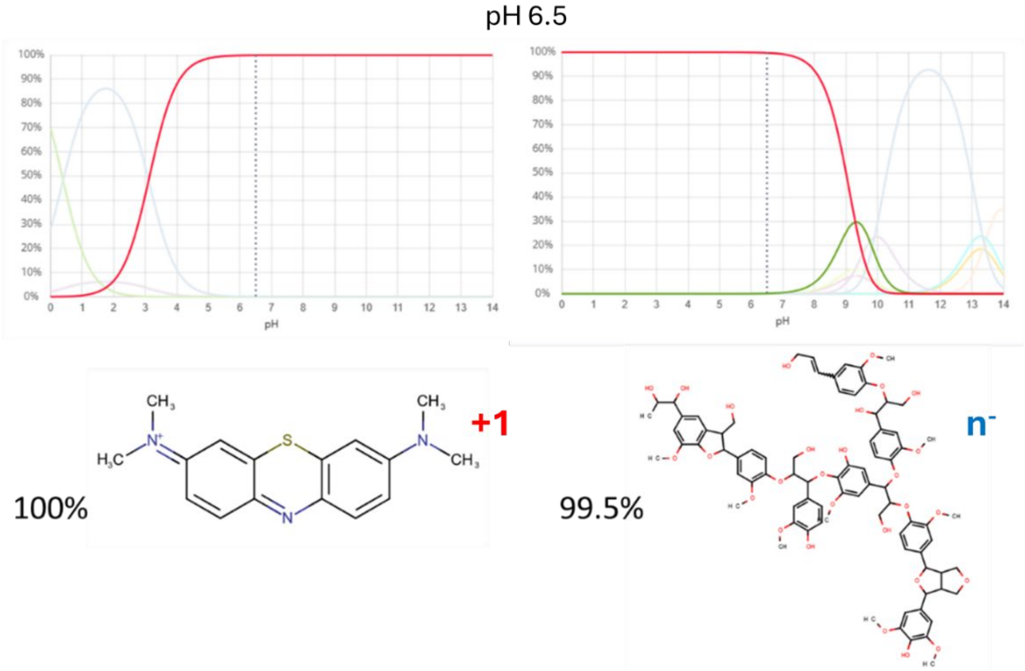

Figure S6. Lignin and Methylene Blue microspecies distribution at pH 6.5 (https://playground.calculators.cxn.io/)

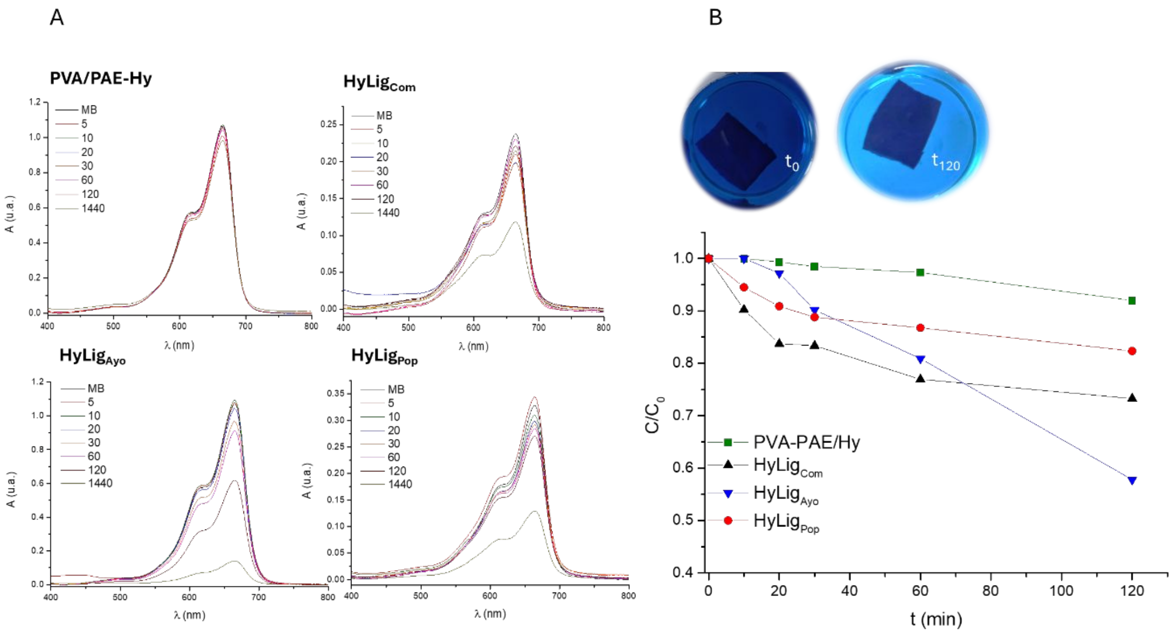

Figure S7. A) adsorption spectra of MB at different times on different hydrogels; B) MB removal rates with different hydrogels.

Table S2. The previous reported hydrogel composite for the MB adsorption

**Name of sample**	**Adsorption capacity mg/g**	**References**
Sulfomethylated lignin/polyacrylic acid hydrogel composite	777.1	[1]
Lignin/sodium alginate hydrogel	388.8	[2]
Lignin/sodium alginate hydrogel beads	254.3	[3]
Bentonite-doped lignin hydrogel spheres	1047.7	[4]
Carboxylated lignin/ alginate hybrid beads	613.0	[5]
Cellulose nanofibrils/alkali lignin/montmorillonite/polyvinyl alcohol hydrogel	67.2	[6]
Lignosulfonate-*graft*-poly[acrylamide-*co*-(acrylic acid)] microgel	154.0	[7]
Lignin/polyvinyl alcohol hydrogel	179.0	[8]
Lignin/hydroxyethyl cellulose/polyvinyl alcohol hydrogels	196.0	[9]
Lignin derivate magnetic hydrogel microspheres	43.0	[10]

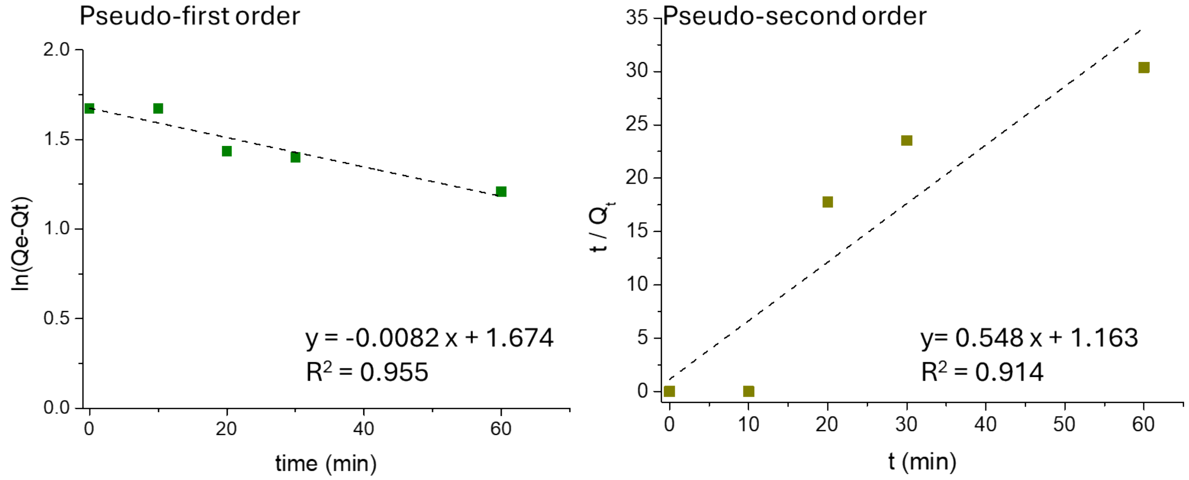

Figure S8. Kinetics studies of PVA/PAE-Hy: Pseudo-first-order and Pseudo-second-order kinetic model

Table S3. Linear fits result for pseudo-first order and Pseudo-second order models.

**Pseudo-second order**	**Linear fit**	R^2
**PVA/PAE-Hy**	y= 0.548 x + 1.163	0.914
**HyLyg$_{Com}$***	y = 0.329 x - 2.270	0.956
**HyLyg$_{Ayo}$***	y = -0.039x + 5.320 (silvia)<br>y = 0.016x + 3.900 (mio)	0.341<br>0.909
**HyLyg$_{Pop}$***	y = 0.083 x + 1.482	0.974

Pseudo-first order	Linear fit	R^2
**PVA/PAE-Hy**	y = -0.0082 x + 1.674	0.955
**HyLyg$_{Com}$***	y = 5.71 10^{-4} x +3.220	0.151
**HyLyg$_{Ayo}$***	y = 0.0585 x + 4.051	0.978
**HyLyg$_{Pop}$***	y = 0.0025 x + 3.560	0.917

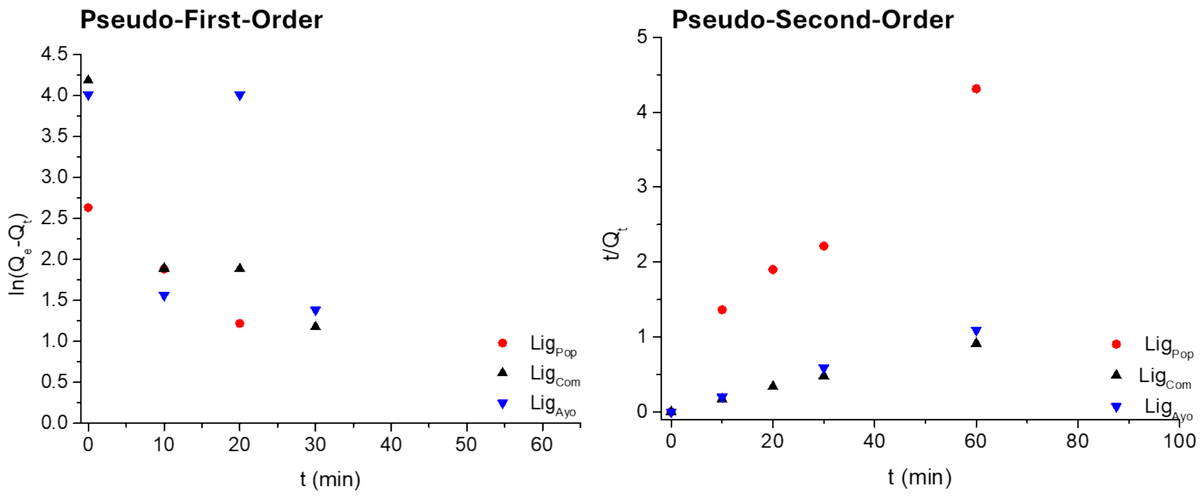

Figure S9. Kinetics studies of pristine lignin (Lig$_{Com}$, Lig$_{Pop}$, Lig$_{Ayo}$): Pseudo-first-order and Pseudo-second-order kinetic model

Table S4. Calculated parameters for kinetic models and intraparticle diffusion models of MB on Lignin powder

Model	Parameters	Lyg$_{Com*}$	Lyg$_{Ayo*}$	Lyg$_{Pop*}$
Pseudo-1st order	k_1 x 10^{-2} (min^{-1})	20.72	12.73	26.84
	q_{cal} (mg/g)	38.10	148.01	18.62
	R^2	0.890	0.918	0.952
Pseudo-2nd order	k_2 x 10^{-2} (g/(mg min))	1.30	2.13	1.66
	q_{cal} (mg/g)	66.22	55.25	30.80
	R^2	0.999	0.999	0.990

Table S5. The comparison of kinetic parameters for various lignin-based adsorbents

**Model**	**Parameters**	**L-F/M** [11]	**Fe-LB** [12]	**ALiCE** [13]
Pseudo-1st order	k_1 x 10^{-2} (min^{-1})	1.61	25.10	0.30
	q_{cal} (mg/g)	83.93	112.17	20.70
	R^2	0.970	0.886	0.977
Pseudo-2nd order	k_2 x 10^{-2} (g/(mg min))	0.0018	0.55	0.020 10^2
	q_{cal} (mg/g)	99.97	202.8	16.36
	R^2	0.99	0.999	0.993
Intra particle diffusion	K_{dI} (mg/g $min^{1/2}$)	1.51	31.74	1.046
	C (mg/g)	-4.18	78.85	---
	R^2	0.980	0.966	---
L-F/M = lignin-Fe/Mn binary oxide blend nanocomposite; Fe/LB = Fe-modified lignin-based biochar; ALiCE = activated lignin-chitosan extruded				

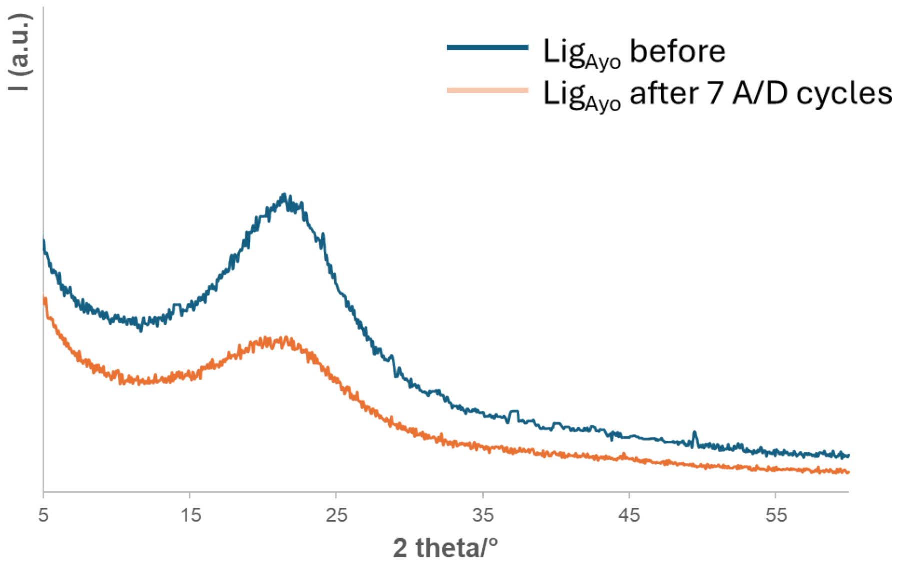

Figure S10. Diffraction patterns of Lig_{Ayo} collected before and after 7 absorption/desorption cycles

References

[1] S.-F. Sun, H.-F. Wan, X. Zhao, C. Gao, L.-P. Xiao, R.-C. Sun Facile construction of lignin-based network composite hydrogel for efficient adsorption of methylene blue from wastewater Int. J. Biol. Macromol., 253 (2023), 126688.

[2] C. Wang, X. Feng, S. Shang, H. Liu, Z. Song, H. Zhang Lignin/sodium alginate hydrogel for efficient removal of methylene blue Int. J. Biol. Macromol., 237 (2023), 124200.

[3] T. Chen, H. Liu, J. Gao, G. Hu, Y. Zhao, X. Tang, X. Han Efficient removal of methylene blue by bio-based sodium alginate/lignin composite hydrogel beads Polymers, 14 (14) (2022), 2917.

[4] M. Jiang, N. Niu, L. Chen A template synthesized strategy on bentonite-doped lignin hydrogel spheres for organic dyes removal Sep. Purif. Technol., 285 (2022), 120376.

[5] J.-C. Kim, J. Kim, J. Park, J.-K. Oh, I.-G. Choi, H.W. Kwak Highly efficient and sustainable alginate/carboxylated lignin hybrid beads as adsorbent for cationic dye removal React. Funct. Polym., 161 (2021), 104839.

[6] J. Luo, X. Ma, X. Zhou, Y. Xu Construction of physically crosslinked cellulose nanofibrils/alkali lignin/montmorillonoite/polyvinyl alcohol network hydrogel and its application in methylene blue removal Cellulose, 28 (9) (2021), 5531-5543.

[7] D. Yiamsawas, W. Kangwansupamonkon, S. Kiatkamjornwong Lignin-based microgels by inverse suspension polymerization: syntheses and dye removal Macromol. Chem. Phys., 222 (23) (2021), 2100285.

[8] L. Wu, S. Huang, J. Zheng, Z. Qiu, X. Lin, Y. Qin Synthesis and characterization of biomass lignin-based PVA super-absorbent hydrogel Int. J. Biol. Macromol., 140 (2019), 538-545

[9] S. Huang, L. Wu, T. Li, D. Xu, X. Lin, C. Wu Facile preparation of biomass lignin-based hydroxyethyl cellulose super-absorbent hydrogel for dye pollutant removal Int. J. Biol. Macromol., 137 (2019), 939-947.

[10] Y. Meng, C. Li, X. Liu, J. Lu, Y. Cheng, L.-P. Xiao, H. Wang Preparation of magnetic hydrogel microspheres of lignin derivate for application in water Sci. Total Environ., 685 (2019), 847-855.

[11] Yu, H.; Yang, J.; Shi, P.; Li, M.; Bian, J. Synthesis of a Lignin-Fe/Mn Binary Oxide Blend Nanocomposite and Its Adsorption Capacity for Methylene Blue. *ACS Omega* , *6* (26) (2021) 16837–16846. https://doi.org/10.1021/acsomega.1c01405.

[12] Sun, Y.; Wang, T.; Han, C.; Lv, X.; Bai, L.; Sun, X.; Zhang, P. Facile Synthesis of Fe-Modified Lignin-

Based Biochar for Ultra-Fast Adsorption of Methylene Blue: Selective Adsorption and Mechanism Studies. Bioresour. Technol. 344 (2022) 126186. https://doi.org/10.1016/j.biortech.2021.126186.

[13] Apostol, I.; Anghel, N.; Doroftei, F.; Bele, A.; Spiridon, I. Xanthan or Esterified Xanthan/Cobalt Ferrite-Lignin Hybrid Materials for Methyl Blue and Basic Fuchsine Dyes Removal: Equilibrium, Kinetic and Thermodynamic Studies. Mater. Today Chem. 27 (2023) 101299. https://doi.org/10.1016/j.mtchem.2022.101299.
